# Long-term survival after mitral valve surgery for post-myocardial infarction papillary muscle rupture

**DOI:** 10.1186/s13019-015-0213-1

**Published:** 2015-01-27

**Authors:** Wobbe Bouma, Inez J Wijdh-den Hamer, Bart M Koene, Michiel Kuijpers, Ehsan Natour, Michiel E Erasmus, Jayant S Jainandunsing, Iwan CC van der Horst, Joseph H Gorman, Robert C Gorman, Massimo A Mariani

**Affiliations:** 1 Department of Cardiothoracic Surgery, University of Groningen, University Medical Center Groningen, Groningen, the Netherlands; 2 Department of Anesthesiology and Pain Medicine, University of Groningen, University Medical Center Groningen, Groningen, the Netherlands; 3 Department of Critical Care, University of Groningen, University Medical Center Groningen, Groningen, the Netherlands; 4 Gorman Cardiovascular Research Group, University of Pennsylvania, Hospital of the University of Pennsylvania, Philadelphia, PA USA

**Keywords:** Myocardial infarction, Papillary muscle (rupture), Mitral regurgitation, Mitral valve repair, Mitral valve replacement, Outcome

## Abstract

**Background:**

Papillary muscle rupture (PMR) is a rare, but dramatic mechanical complication of myocardial infarction (MI), which can lead to rapid clinical deterioration and death. Immediate surgical intervention is considered the optimal and most rational treatment, despite high risks. In this study we sought to identify overall long-term survival and its predictors for patients who underwent mitral valve surgery for post-MI PMR.

**Methods:**

Fifty consecutive patients (mean age 64.7 ± 10.8 years) underwent mitral valve repair (n = 10) or replacement (n = 40) for post-MI PMR from January 1990 through May 2014. Clinical data, echocardiographic data, catheterization data, and surgical data were stored in a dedicated database. Follow-up was obtained in June of 2014; mean follow-up was 7.1 ± 6.8 years (range 0.0-22.2 years). Univariate and multivariate Cox proportional hazard regression analyses were performed to identify predictors of long-term survival. Kaplan-Meier curves were compared with the log-rank test.

**Results:**

Kaplan-Meier cumulative survival at 1, 5, 10, 15, and 20 years was 71.9 ± 6.4%, 65.1 ± 6.9%, 49.5 ± 7.6%, 36.1 ± 8.0% and 23.7 ± 9.2%, respectively. Univariate and multivariate analyses revealed logistic EuroSCORE ≥40% and EuroSCORE II ≥25% as strong independent predictors of a lower overall long-term survival. After removal of the EuroSCOREs from the model, preoperative inotropic drug support and mitral valve replacement (MVR) without (partial or complete) preservation of the subvalvular apparatus were independent predictors of a lower overall long-term survival.

**Conclusions:**

Logistic EuroSCORE ≥40%, EuroSCORE II ≥25%, preoperative inotropic drug support and MVR without (partial or complete) preservation of the subvalvular apparatus are strong independent predictors of a lower overall long-term survival in patients undergoing mitral valve surgery for post-MI PMR. Whenever possible, the subvalvular apparatus should be preserved in these patients.

## Background

In the current era of early reperfusion with primary percutaneous coronary intervention (PCI) following acute ST-elevation myocardial infarction (STEMI), the incidence of post-myocardial infarction papillary muscle rupture (post-MI PMR) has dropped from 1-5% in the eighties and early nineties to <0.5% in recent years [1-3]. Although rare, PMR is still a dramatic complication, which can lead to rapid clinical deterioration and death [1]. Approximately 80% of ruptures occur within 7 days after MI, but a delayed rupture several weeks or months after MI is also possible [1,2,4]. The natural history of post-MI PMR is extremely unfavorable and under medical treatment alone mortality may be as high as 50% in the first 24 hours (especially when PMR is complete), and as high as 80% in the first week [4,5].

Since the first mitral valve replacement (MVR) for post-MI PMR in 1965 [6], several reports have emphasized that immediate surgical intervention is the optimal and most rational treatment for acute PMR, despite high risks [2,4,7]. Although mitral valve repair may improve outcome due to a better preservation of postoperative left ventricular (LV) function [8-13], MVR is generally preferred in these haemodynamically unstable, high-risk patients [7,14-18].

In this study we sought to identify overall long-term survival and its predictors for patients who underwent mitral valve surgery for post-MI PMR.

## Methods

This study was conducted in accordance with the guidelines of the University Medical Center Groningen Institutional Review Board.

### Patients

Between January 1990 and May 2014, 50 consecutive patients underwent mitral valve surgery for moderate-to-severe (grade 3+) or severe (grade 4+) mitral regurgitation (MR) caused by post-MI PMR. Clinical data, echocardiographic data, catheterization data, and surgical data were stored in a dedicated database. Baseline patient characteristics are summarized in Table 1. All patients had a documented MI before PMR. Infarct location was determined electrocardiographically and echocardiographically (by the detection of wall motion abnormalities). Four patients (8%) had a history of congestive heart failure, 12 patients (24%) had hypertension, 9 patients (18%) had diabetes mellitus, 16 patients (32%) were smokers, 6 patients (12%) had hypercholesterolemia, 7 patients (14%) were obese (body mass index > 30 kg/m^2^), 2 patients (4%) had peripheral vascular disease, 7 patients (14%) had a family history of coronary artery disease, 1 patient (2%) had chronic renal disease, 3 patients (6%) had chronic obstructive pulmonary disease, and 5 patients (10%) had cerebrovascular disease.

**Table 1:** **Preoperative patient data (n = 50)**

Variable^a^		Value
Age, years		64.7 ± 10.8
Gender		
Male		36 (72)
Female		14 (28)
NYHA functional class		
Class III		7 (14)
Class IV		43 (86)
EuroSCORE I (logistic), %		29.9 ± 22.6
EuroSCORE II, %		19.6 ± 14.5
Previous myocardial infarction		50 (100)
Inferior and/or posterior		34 (68)
Inferoposterolateral		13 (26)
Anterolateral		9 (18)
Coronary artery disease		50 (100)
Left main stenosis		3 (6)
One-vessel disease		24 (48)
Two-vessel disease		18 (36)
Three-vessel disease		8 (16)
Infarct related artery		
Left anterior descending coronary artery		1 (2)
Left circumflex coronary artery		29 (58)
Right coronary artery		20 (40)
Previous percutaneous coronary intervention		14 (28)
Previous cardiac surgery		0 (0)
Preoperative grade of mitral regurgitation		
3+ (moderate)		1 (2)
4+ (severe)		49 (98)
Preoperative LV function		
Normal	(EF >50%)	34 (68)
Moderately impaired	(EF 30-50%)	11 (22)
Severely impaired	(EF <30%)	5 (10)
Heart rhythm		
Sinus rhythm		45 (90)
Atrial fibrillation		5 (10)
Pacemaker		0 (0)
Pulmonary artery pressure		
Systolic/diastolic, mmHg		45 ± 13/25 ± 10
Mean, mmHg		32 ± 10
Pulmonary capillary wedge pressure, mmHg		24 ± 14
Mechanical ventilation		23 (46)
Inotropic drug support		28 (56)
Intra-aortic balloon pump		23 (46)
Serum creatinine, μmol/L		159 ± 94
Acute renal failure		10 (20)
Cardiogenic shock		33 (66)

^a^Data are presented as mean ± standard deviation or number (%).

EF = ejection fraction; LV = left ventricle; NYHA = New York Heart Association.

### Echocardiography and coronary angiography

All patients underwent preoperative echocardiography (transthoracic (TTE) and/or transesophageal (TEE)) and coronary angiography. TTE accurately revealed the diagnosis of PMR in 20 patients (40%). PMR was suspected in the remaining 30 patients (60%) and confirmed with TEE in 24 patients (48%). In 6 patients (12%) the exact mechanism of MR remained inconclusive. Left ventricular function was assessed by echocardiography. In addition, wall motion abnormalities were documented for infarct localization.

### Surgical technique

Surgical data is summarized in Table 2.

**Table 2:** **Surgical data (n = 50)**

Variable^a^	Value
Mitral valve surgery	
Salvage	2 (4)
Emergent	31 (62)
Urgent	11 (22)
Elective	6 (12)
Timing of mitral valve surgery	
Surgery ≤7 days after MI	29 (58)
Surgery >7 days and ≤30 days after MI	9 (18)
Surgery >30 days after MI	12 (24)
Mitral valve replacement	40 (80)
Mechanical prosthesis	36 (90)
Bioprosthesis	4 (10)
Preservation of the SA (partial or complete)	26 (65)
AMVL and attached SA (partial or complete)	5 (19)
PMVL and attached SA (partial or complete)	14 (54)
AMVL and PMVL and attached SA (partial or complete)	7 (27)
Mitral valve repair	10 (20)
Concomitant surgery	28 (56)
Coronary artery bypass grafting	24 (48)
Septal rupture closure	2 (4)
Aortic valve replacement	1 (2)
Tricuspid valve plasty	2 (4)
Duration of surgery, min	278 ± 88
Cardiopulmonary bypass time, min	179 ± 67
Aortic cross-clamp time, min	98 ± 35
Intraoperative IABP requirement	26 (52)

^a^Data are presented as mean ± standard deviation or number (%).

AMVL = anterior mitral valve leaflet; IABP = intra-aortic balloon pump; MI = myocardial infarction; PMVL = posterior mitral valve leaflet; SA = subvalvular apparatus.

Patients were considered to undergo a salvage operation when brought to the operating room under cardiopulmonary resuscitation, an emergency operation when brought to the operating room directly from the catheterization lab or intensive care unit because of haemodynamic instability, and an urgent operation when operated on during the same hospitalization as for angiography because their discharge was deemed medically unreasonable [19]. Otherwise the operation was considered elective.

PMR was confirmed during surgery in all patients. When a papillary muscle (PM) was divided into several heads, rupture of a single head was defined as “partial” [11,12]. In case of detachment of the main insertion of a head which still remained fixed to the remnant PM via muscular bridges, rupture was defined as “incomplete” [11,12]. Rupture of the whole PM was defined as “total and complete” [11,12]. Posteromedian papillary muscle rupture (PMPMR) occurred in 44 patients (88%), anterolateral papillary muscle rupture (ALPMR) occurred in 5 patients (10%), and combined complete PMPMR and ALPMR occurred in 1 patient (2%). Complete PMPMR occurred in 15 patients (39%), incomplete PMPMR occurred in 2 patients (5%), and partial PMPMR occurred in 25 patients (57%). Complete ALPMR occured in 4 patients (80%), incomplete ALPMR did not occur, and partial ALPMR occurred in 1 patient (20%). Isolated posterior mitral valve leaflet (PMVL) prolapse was found in 15 patients (30%), isolated anterior mitral valve leaflet (AMVL) prolapse was found in 12 patients (24%), and combined prolapse was found in 23 patients (46%).

Myocardial protection was carried out using moderate systemic hypothermia and antegrade or combined antegrade and retrograde cardioplegia. The mitral valve was exposed with a left atriotomy (in 36 patients), with a transseptal approach (in 13 patients), or with a left ventriculotomy (in one patient with a ventricular septal rupture). Surgeon’s choice dictated treatment strategy. Ten patients underwent mitral valve repair (20%). The mitral valve was repaired by reimplantation of the PM in the LV wall combined with an annuloplasty ring in 1 patient, by reimplantation of the PM in the corresponding PM with a sandwiched pledget-reinforced polytetrafluorethylene (PTFE) suture combined with an annuloplasty ring in 2 patients, by quadrangular resection of P2 combined with an annuloplasty ring in 6 patients, and by commissuroplasty combined with an annuloplasty ring in 1 patient. Mitral valve repair with a quadrangular resection of P2 combined with an annuloplasty ring failed intraoperatively in 1 patient and resulted in MVR. Concomitant procedures were performed in 28 patients (56%) and concomitant coronary artery bypass grafting (CABG) was performed in 24 patients (48%) (Table 2). After weaning from cardiopulmonary bypass mitral valve competence was confirmed with TEE.

Patients who underwent mitral valve repair or MVR with a bioprosthesis received acenocoumarol treatment for 3 months and patients who underwent MVR with a mechanical prosthesis were put on lifelong acenocoumarol treatment. In addition, patients who underwent concomitant CABG also received lifelong acetylsalicylic acid treatment.

### Follow-up

Follow-up was obtained in June of 2014 directly from outpatient visits or by telephone interview with the patient and/or the referring physician. No patient was lost to follow-up. Overall long-term mortality was defined as death of any cause after surgery (and included in-hospital deaths).

### Statistics

Continuous variables were expressed as mean ± standard deviation. Categorical variables were expressed as percentages.

Cox proportional-hazard regression analysis was used to determine univariate predictors and multivariate independent predictors of overall long-term survival. Univariate variables with *P* < 0.10 were included in the multivariate analysis. Age and gender were included in all multivariate models, irrespective of the results of univariate analysis. Multivariate analysis was performed with the logistic EuroSCORE (continuous variable) (model 1), with the EuroSCORE II (continuous variable) (model 2), with logistic EuroSCORE ≥40% (categorical variable) (model 3), with EuroSCORE II ≥25% (categorical variable) (model 4), and without the EuroSCOREs (model 5). Multivariate Cox proportional-hazard regression analysis by means of a forward stepwise algorithm (cutoff for entry and removal set at a significance level of 0.05) was performed to identify independent predictors of overall long-term survival. Hazard ratios were reported with 95% confidence intervals (CI). Goodness of fit of the final model was assessed with the Chi square (*χ*^2^) goodness-of-fit test (the log likelihood statistic).

Separate multivariate analyses were performed with a cutoff value of 40% for the logistic EuroSCORE (model 3) and a cutoff value of 25% for the EuroSCORE II (model 4). We have previously shown that these values were optimal cutoff values in predicting in-hospital mortality [13]. The EuroSCOREs were included as categorical variables at these cutoff values to determine if they could also predict overall long-term survival.

Survival curves were calculated and presented according to the product-limit method of Kaplan and Meier. Cumulative survival was expressed as percentage ± standard error. Survival was expressed as mean ± standard error. Differences in survival between groups were compared with the log-rank test.

All calculations were performed using a commercially available statistical package (IBM SPSS Statistics 21.0; IBM Corporation, Chicago, IL, USA). Statistically significant differences were established at *P* < 0.05.

## Results

### Overall long-term survival

**Figure 1:**
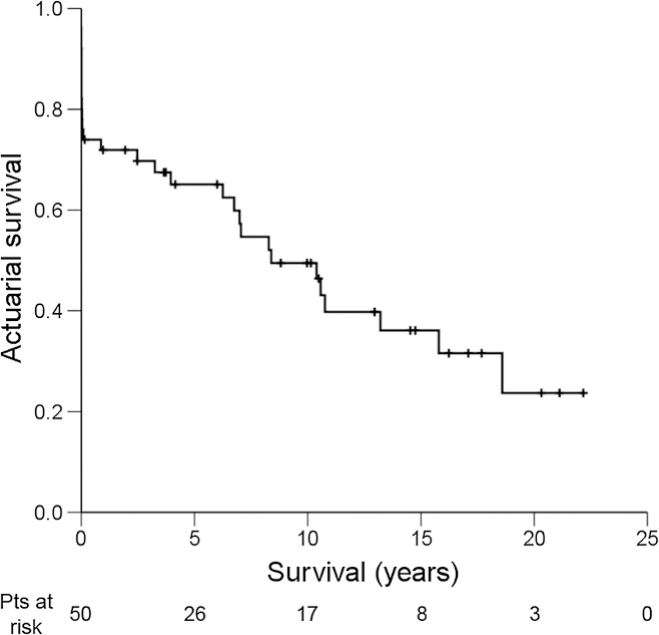
**Kaplan-Meier actuarial overall long-term survival after mitral valve surgery for post-MI PMR.** Pts = patients; + = censored.

Mean follow up was 7.1 ± 6.8 years (range: 0.0 to 22.2 years). Total follow-up was 353.1 patient-years. Actuarial overall long-term survival after mitral valve surgery for post-MI PMR is shown in Figure 1. Kaplan-Meier cumulative survival at 1, 5, 10, 15, and 20 years was 71.9 ± 6.4%, 65.1 ± 6.9%, 49.5 ± 7.6%, 36.1 ± 8.0% and 23.7 ± 9.2%, respectively. Causes of death are shown in Table 3. At last follow-up all survivors (n = 21) were in NYHA functional class I or II.

**Table 3:** **Postoperative patient data (n = 50)**

Variable/Condition^a^	Value
Reoperation for recurrent MR	1 (2)
Causes of death (n = 29)	
(End-stage) heart failure	9 (31)
Refractory cardiogenic shock	3 (10)
Haemorrhagic shock (massive bleeding)	2 (7)
Acute myocardial infarction	1 (3)
Arrhythmic sudden death	1 (3)
Septal rupture	1 (3)
Left ventricular rupture	1 (3)
Ruptured aortic aneurysm	1 (3)
Non-cardiac	3 (10)
Unknown	7 (24)

^a^Data are presented as number (%).

MR = mitral regurgitation.

### Predictors of overall long-term survival

Univariate and multivariate Cox regression analyses of long-term survival are shown in Table 4. Multivariate analysis was performed with the logistic EuroSCORE (continuous variable) (model 1), with the EuroSCORE II (continuous variable) (model 2), with logistic EuroSCORE ≥40% (categorical variable) (model 3), with EuroSCORE II ≥25% (categorical variable) (model 4), and without the EuroSCOREs (model 5).

**Table 4:** **Predictors of a lower overall long-term survival by univariate and multivariate Cox proportional hazard regression analysis**

	Univariate analysis			Multivariate analysis		
Variable	HR	95% CI	P value	HR	95% CI	P value
Age, y	1.07	(1.02-1.11)	0.003	−	−	−
Logistic EuroSCORE, %	1.07	(1.04-1.09)	<0.001	1.06	(1.03-1.09)	<0.001^a^
EuroSCORE II, %	1.07	(1.04-1.10)	<0.001	1.06	(1.02-1.09)	0.002^b^
Logistic EuroSCORE ≥40%	14.28	(5.65-36.11)	<0.001	16.69	(4.23-65.84)	<0.001^c^
EuroSCORE II ≥25%	6.16	(2.81-13.48)	<0.001	4.31	(1.67-11.14)	0.003^d^
Preoperative LVEF <30%	4.31	(1.58-11.81)	0.004	−	−	−
PAP (systolic), mmHg	1.03	(1.00-1.06)	0.069	−	−	−
PAP (diastolic), mmHg	1.04	(1.00-1.08)	0.071	−	−	−
Mean PAP, mmHg	1.04	(1.00-1.08)	0.059	−	−	−
Preoperative inotropic drug support	3.51	(1.56-7.90)	0.002	6.27	(1.97-19.95)	0.002^e^
Preoperative IABP requirement	2.69	(1.25-5.77)	0.011	−	−	−
Serum creatinine, μmol/L	1.00	(1.00-1.01)	0.059	−	−	−
Acute renal failure	2.29	(0.96-5.48)	0.062	−	−	−
Cardiogenic shock	3.37	(1.35-8.41)	0.009	−	−	−
Salvage/Emergent mitral valve surgery	2.56	(1.08-6.06)	0.032	−	−	−
MVR without preservation of the subvalvular apparatus	2.60	(1.23-5.46)	0.012	3.40	(1.19-9.69)	0.022^e^
No concomitant CABG	1.89	(0.89-4.01)	0.098	−	−	−
Concomitant septal rupture	4.17	(0.94-18.54)	0.061	−	−	−
Cardiopulmonary bypass time, min	1.01	(1.00-1.01)	0.026	−	−	−
Intraoperative IABP requirement	2.99	(1.37-6.51)	0.006	−	−	−

^a^Model 1; ^b^Model 2; ^c^Model 3; ^d^Model 4; ^e^Model 5.

CABG = coronary artery bypass grafting; CI = confidence interval; HR = hazard ratio; IABP = intra-aortic balloon pump; LVEF = left ventricular ejection fraction; MVR = mitral valve replacement; PAP = pulmonary artery pressure.

#### Model 1

Multivariate Cox proportional-hazard regression analysis with the logistic EuroSCORE as a continous variable revealed the logistic EuroSCORE as an independent predictor of overall long-term survival (hazard ratio 1.06 (95% CI 1.03-1.09), Wald *χ*^2^ 17.84, *P* < 0.001). The *χ*^2^ goodness-of-fit test (log likelihood ratio test) was significant, indicating that this multivariate model is a good fit (*χ*^2^ = 22.57, df = 1, *P* < 0.001).

#### Model 2

Multivariate Cox proportional-hazard regression analysis with the EuroSCORE II as a continuous variable revealed the EuroSCORE II as an independent predictor of overall long-term survival (hazard ratio 1.06 (95% CI 1.02-1.09), Wald *χ*^2^ 9.68, *P* = 0.002). The *χ*^2^ goodness-of-fit test (log likelihood ratio test) was significant, indicating that this multivariate model is a good fit (*χ*^2^ = 10.44, df = 1, *P* = 0.001).

#### Model 3

**Figure 2:**
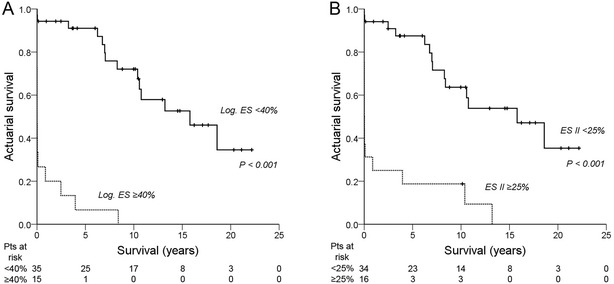
**Kaplan-Meier actuarial overall long-term survival after mitral valve surgery for post-MI PMR. A**: Logistic EuroSCORE <40% vs. Logistic EuroSCORE ≥40%. **B**: EuroSCORE II <25% vs. EuroSCORE ≥25%. ES II = EuroSCORE II; Log. ES = logistic EuroSCORE; Pts = patients; + = censored.

Multivariate Cox proportional-hazard regression analysis with the logistic EuroSCORE ≥40% (categorical variable) revealed the logistic EuroSCORE ≥40% as an independent predictor of a lower overall long-term survival (hazard ratio 16.69 (95% CI 4.23-65.84), Wald *χ*^2^ 16.16, *P* < 0.001). The *χ*^2^ goodness-of-fit test (log likelihood ratio test) was significant, indicating that this multivariate model is a good fit (*χ*^2^ = 26.56, df = 1, *P* < 0.001). The influence of a logistic EuroSCORE ≥40% on overall long-term survival is shown in a Kaplan-Meier survival curve (Figure 2A). Patients with a logistic EuroSCORE ≥40% had a significantly lower mean overall long-term survival (1.1 ± 0.6 years) compared to patients with a logistic EuroSCORE <40% (14.5 ± 1.4 years, *P* < 0.001).

#### Model 4

Multivariate Cox proportional-hazard regression analysis with the logistic EuroSCORE ≥25% (categorical variable) revealed the logistic EuroSCORE ≥25% as an independent predictor of a lower overall long-term survival (hazard ratio 4.31 (95% CI 1.67-11.14), Wald *χ*^2^ 9.12, *P* = 0.003). The *χ*^2^ goodness-of-fit test (log likelihood ratio test) was significant, indicating that this multivariate model is a good fit (*χ*^2^ = 10.64, df = 1, *P* = 0.001). The influence of an EuroSCORE II ≥25% on overall long-term survival is shown in a Kaplan-Meier survival curve (Figure 2B). Patients with an EuroSCORE II ≥25% had a significantly lower mean overall long-term survival (2.5 ± 1.2 years) compared to patients with an EuroSCORE II <25% (14.1 ± 1.5 years, *P* < 0.001).

#### Model 5

**Figure 3:**
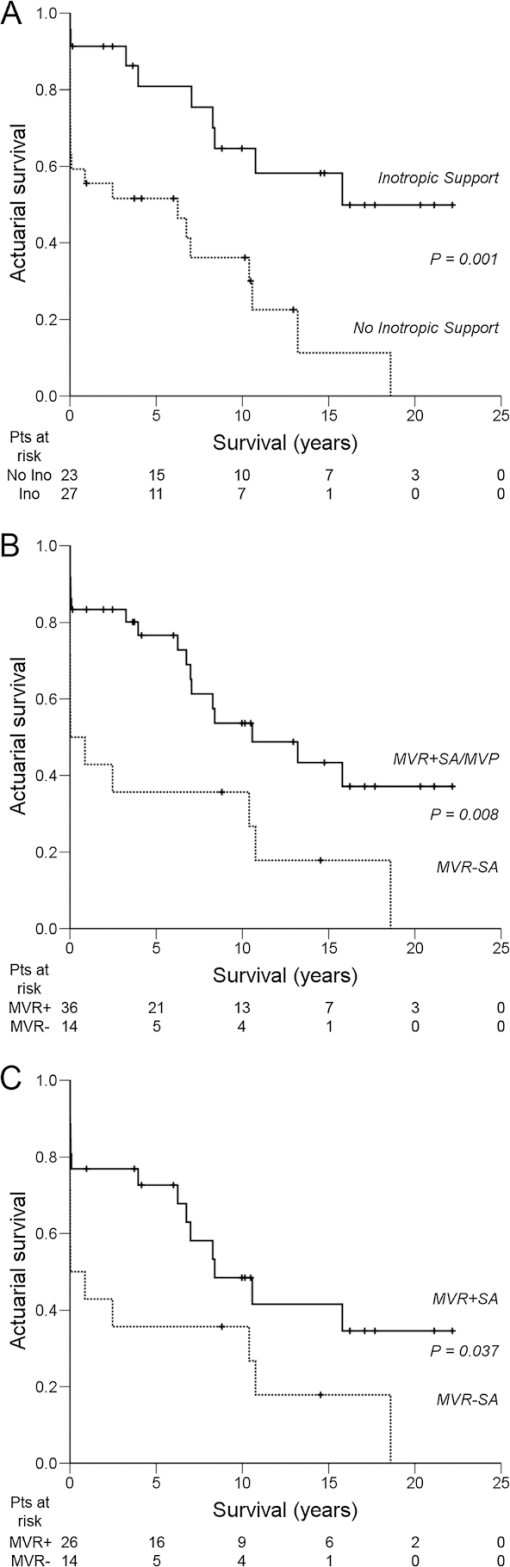
**Kaplan-Meier actuarial overall long-term survival after mitral valve surgery for post-MI PMR. A**: No preoperative inotropic drug support vs. preoperative inotropic drug support. **B**: Mitral valve replacement with (partial or complete) preservation of the subvalvular apparatus or mitral valve repair vs. mitral valve replacement without (partial or complete) preservation of the subvalvular apparatus. **C**: Mitral valve replacement with (partial or complete) preservation of the subvalvular apparatus vs. mitral valve replacement without (partial or complete) preservation of the subvalvular apparatus. MVP = mitral valve plasty/repair; MVR = mitral valve replacement; SA = subvalvular apparatus; Pts = patients; + = censored.

After removal of the EuroSCOREs from the model, preoperative inotropic drug support (hazard ratio 6.27 (95% CI 1.97-19.95), Wald *χ*^2^ 9.64, *P* = 0.002) and MVR without (partial or complete) preservation of the subvalvular apparatus (SA) (hazard ratio 3.40 (95% CI 1.19-9.69), Wald *χ*^2^ 5.21, *P* = 0.022) were independent predictors of a lower overall long-term survival. The *χ*^2^ goodness–of-fit test (log likelihood ratio test) was significant, indicating that this multivariate model is a good fit (*χ*^2^ = 14.06, df = 2, *P* = 0.001). The influence of preoperative inotropic drug support and preservation of the SA on overall long-term survival are shown in Kaplan-Meier survival curves (Figure 3A-C). Patients who required preoperative inotropic drug support had a significantly lower mean overall long-term survival (6.2 ± 1.4 years) compared to patients who did not require preoperative inotropic drug support (14.7 ± 1.9 years, *P* = 0.001) (Figure 3A). Patients in which the mitral valvular-ventricular continuity was preserved (i.e. patients who underwent mitral valve repair or MVR with (partial or complete) preservation of the SA) had a significantly higher mean overall long-term survival (12.4 ± 1.6 years) compared to patients who underwent MVR without (partial or complete) preservation of the SA (5.4 ± 2.1 years, *P* = 0.008) (Figure 3B). A survival comparison between patients who underwent MVR with (n = 26) or without (partial or complete) preservation of the SA (n = 14) also showed that MVR with (partial or complete) preservation of the SA carries a significantly higher mean overall long-term survival (11.4 ± 1.9 years) than MVR without (partial or complete) preservation of the SA (5.4 ± 2.1 years, *P* = 0.037) (Figure 3C). The type of preservation (partial or complete preservation of the anterior, or posterior, or both mitral valve leaflets and the attached SA) did not influence overall long-term survival.

Univariate and multivariate analyses showed that both MVR and concomitant CABG did not predict overall long-term survival. Patients who underwent MVR had a mean overall long-term survival (9.2 ± 1.5 years) that did not differ significantly from the mean overall long-term survival in patients who underwent mitral valve repair (14.0 ± 2.7 years, *P* = 0.111). Patients who did not undergo concomitant CABG had a mean overall long-term survival (8.1 ± 1.5 years) that did not differ significantly from the mean overall long-term survival in patients who did undergo concomitant CABG (12.7 ± 2.1 years, *P* = 0.089).

## Discussion

Overall long-term survival for patients who underwent mitral valve surgery for post-MI PMR was 71.9 ± 6.4% after 1 year, 65.1 ± 6.9% after 5 years, 49.5 ± 7.6% after 10 years, 36.1 ± 8.0% after 15 years, and 23.7 ± 9.2% after 20 years in this study. Previous studies reported similar overall long-term survival rates with 1 year-survival ranging between 75% and 81% [14,15], 5 year-survival ranging between 65% and 68% [14,15,17] and 10 year-survival ranging between 32% and 56% [14,15,17].

Both the logistic EuroSCORE and EuroSCORE II were strong independent predictors of overall long-term survival in this study. The logistic EuroSCORE was introduced in 1999 and has proven its value as an important risk-stratification model in cardiac surgery [20]. However, the model was mainly designed for predicting in-hospital mortality in CABG patients and its predictive power has declined in recent years [21]. To more accurately predict in-hospital mortality for patients undergoing a wider range of cardiac surgical procedures the EuroSCORE II was introduced in 2012 [22]. Although both EuroSCOREs were not specifically designed for patients undergoing mitral valve surgery for post-MI PMR or to assess long-term survival, these models can both be used as predictors of overall long-term survival in this setting. Both EuroSCOREs contain items that were shown to be univariate predictors (*P* < 0.10) of overall long-term survival in this study, such as age, serum creatinine (incorporated in the creatinine clearance in EuroSCORE II), LVEF, (systolic) pulmonary artery pressure, preoperative inotropic drug support/intra-aortic balloon pump (IABP) requirement/acute renal failure (all incorporated in the “critical preoperative state” item of both EuroSCOREs), emergency surgery, and postinfarct septal rupture (only incorporated in the logistic EuroSCORE as a separate item) (Table 4). A logistic EuroSCORE ≥40% and an EuroSCORE II ≥25% are both strong independent predictors of a worse overall long-term survival after mitral valve surgery for post-MI PMR (hazard ratio 16.69 and 4.31, respectively).

Preoperative haemodynamic instability and cardiogenic shock following post-MI PMR may warrant the use of inotropes and/or an IABP. However, stabilization should never lead to delay of surgery, because rapid clinical deterioration and death are always imminent [2,4,7]. We previously showed that intraoperative IABP requirement was an independent predictor of in-hospital mortality after mitral valve surgery for post-MI PMR [13], but it was not an independent predictor of overall long-term mortality in this study. Preoperative inotropic drug support was a strong independent predictor of overall long-term survival in this study. Mean overall long-term survival was significantly lower in the group that required preoperative inotropes (6.2 ± 1.4 years) compared to the group that did not require preoperative inotropes (14.7 ± 1.9 years, *P* = 0.001). Preoperative inotropic drug requirement may reflect reduced viability of myocardial reserve and a tendency towards a faster postoperative deterioration of LVEF (and a reduced survival) over time. However, relative preservation of LVEF in most post-MI PMR patients requiring mitral valve surgery [7,14] contradicts this explanation. Alternatively, inotropic drugs themselves may also have deleterious effects, which may only become apparent after a certain period of time. Several studies have shown that inotropic stimulation of contractility in hibernating myocardium without restoration of blood flow increases short-term myocardial contractility at the expense of accelerated apoptosis and further degeneration of myocardial function [23-25]. These effects may also occur after short-term use of inotropic drugs. However, the influence of (preoperative) inotropic drug support on postoperative LVEF was not assessed in this study.

MR secondary to partial or incomplete PMR with limited adjacent tissue damage is often amenable to a reliable repair, provided established repair techniques are used and adjacent tissue is not friable [7,8,10-13,17]. All 10 repair patients in this study experienced partial or incomplete PMR. Complete post-MI PMR generally requires MVR because of friable infarcted tissue [7,11,13,15,17]. In this study mean overall long-term survival in the repair group (14.0 ± 2.7 years) did not differ significantly from the mean overall long-term survival in the replacement group (9.2 ± 1.5 years, *P* = 0.111). A major finding in this study is that (partial or complete) preservation of the SA in patients undergoing mitral valve surgery for post-MI PMR independently predicts and significantly improves overall long-term survival. Patients in which mitral valvular-ventricular continuity or papillary muscle-annular continuity was preserved (i.e. patients who underwent mitral valve repair or MVR with (partial or complete) preservation of the SA) had a significantly higher mean overall long-term survival (12.4 ± 1.6 years) compared to patients who underwent MVR without (partial or complete) preservation of the SA (5.4 ± 2.1 years, *P* = 0.008). A separate survival comparison in the replacement group also showed that mean overall long-term survival is significantly higher after MVR with (partial or complete) preservation of the SA (11.4 ± 1.9 years) compared to MVR without (partial or complete) preservation of the SA (5.4 ± 2.1 years, *P* = 0.037). In general it has been shown that MVR with preservation of the SA maintains postoperative LV contractile function and improves outcome [9]. This study shows that MVR with (partial or complete) preservation of the SA improves overall long-term survival specifically for patients with post-MI PMR. Several techniques are available for preservation of the SA in MVR [26]. The type of preservation (partial or complete preservation of the anterior, or posterior, or both mitral valve leaflets and the attached SA) did not influence overall long-term survival in this study. Although we prefer to preserve as much of the SA in MVR as possible to maintain optimal valvular-ventricular (or papillary muscle-annular) continuity [27], resection of the anterior mitral valve leaflet and the attached SA are frequently required to facilitate implantation of a suitably sized valve, to prevent interference of the SA with the prosthetic valve mechanism, and to prevent left ventricular outflow tract obstruction [26].

The relative importance of concomitant CABG in the setting of mitral valve surgery for post-MI PMR remains unclear. It has been shown that concomitant CABG can improve immediate [7,16] and long-term survival [7] after post-MI PMR, but a more recent study was unable to corroborate those findings for long-term survival [17]. Concomitant CABG was not a predictor of overall long-term survival in this study. Mean overall long-term survival was 12.7 ± 2.1 years in the concomitant CABG group and 8.1 ± 1.5 years in the no concomitant CABG group, *P* = 0.089. Revascularization of any kind (preoperative PCI (balloon angioplasty with or without stenting) (n = 10), concomitant CABG (n = 20), or both (n = 4)) also did not improve overall long-term survival. Mean overall long-term survival was 11.8 ± 1.8 years in the revascularization group and 7.8 ± 1.9 years in the no revascularization group, *P* = 0.143. At this point, there are no randomized studies to determine the relative importance of concomitant CABG or hybrid PCI approaches in the setting of post-MI PMR. Results from the SHOCK (should we emergently revascularize occluded coronaries in cardiogenic shock) trial did show improved long-term survival with early revascularization for cardiogenic shock complicating MI [28], but patients with PMR were excluded in this trial. Concomitant CABG may improve postoperative LV function and survival in the setting of post-MI PMR, but these potential benefits have to be weighed against the consequences of prolonging the duration of cardiopulmonary bypass.

A propensity-matched case–control study recently showed that life expectancy may return to normal (i.e. to the life expectancy of patients with a similar MI, but without PMR) for operative survivors (patients who survived the first 30 days after surgery) [17]. Five-year survival for patients with a similar MI but no PMR was 79 ± 4%; 10-year survival was 55 ± 6% [17]. After recalculation results from this study support those findings with a 5-year survival rate of 86 ± 6% and a 10-year survival rate of 65 ± 9% for operative survivors. Return to normal life expectancy provides an additional argument for prompt diagnosis and aggressive surgical treatment of post-MI PMR.

Even though this is one of the largest studies on mitral valve surgery for post-MI PMR, the number of patients is still relatively small. Other limitations include the long time frame and the non-randomized retrospective design. Larger multicenter (preferably randomized) studies are required to more accurately identify independent predictors of short- and long-term outcome and to determine potential outcome benefits of mitral valve repair over replacement for post-MI PMR. However, such studies would be very difficult to conduct and would probably face major ethical issues.

## Conclusions

Our findings indicate that logistic EuroSCORE ≥40%, EuroSCORE II ≥25%, preoperative inotropic drug support and MVR without (partial or complete) preservation of the subvalvular apparatus are strong independent predictors of a lower overall long-term survival in patients undergoing mitral valve surgery for post-MI PMR. Whenever possible, the subvalvular apparatus should be preserved in these patients.

## Abbreviations

ALPM(R): Anterolateral papillary muscle (rupture)
AMVL: Anterior mitral valve leaflet
CABG: Coronary artery bypass grafting
CI: Confidence interval
ES: EuroSCORE
HR: Hazard ratio
IABP: Intra-aortic balloon pump
LV(EF): Left ventricular (ejection fraction)
MI: myocardial infarction
MR: Mitral regurgitation
MVP: Mitral valve plasty/repair
MVR: Mitral valve replacement
NYHA: New-York Heart Association
PAP: Pulmonary artery pressure
PCI: Percutaneous coronary intervention
PMPM(R): Posteromedian papillary muscle (rupture)
PM(R): Papillary muscle (rupture)
PMVL: Posterior mitral valve leaflet
PTFE: Polytetrafluorethylene
SA: Subvalvular apparatus
SE: Standard error
STEMI: ST-elevation myocardial infarction
TEE: Transesophageal echocardiography
TTE: Transthoracic echocardiography
